# Correction: Measuring multi-spatiotemporal scale tourist destination popularity based on text granular computing

**DOI:** 10.1371/journal.pone.0233068

**Published:** 2020-05-05

**Authors:** Chi Yunxian, Li Renjie, Zhao Shuliang, Guo Fenghua

There is an error in affiliation 4 for author Guo Fenghua. The correct affiliation 4 is: Institute of Geographical Sciences, Hebei Academy of Sciences, Shijiazhuang, China.
