# Supplementary material for: Measuring multi-spatiotemporal scale tourist destination popularity based on text granular computing
Source: PLoS One. 2020 Apr 9;15(4):e0228175. doi: 10.1371/journal.pone.0228175 (PMC7145151; doi:10.1371/journal.pone.0228175)
Supplement: S1 File — (PDF) [file pone.0228175.s001.pdf]

WBT18100885
20181005550

微博开放平台商业数据服务合作协议

本协议由于以下双方于 2018 年 10 月 25 日在北京市海淀区签署：

甲方：北京微梦创科网络技术有限公司

地址：北京市海淀区西北旺东路10号院西区8号楼新浪总部大厦

联系人： 陈思思

联系方式： 010-60618125

联系邮箱： sisi8@staff.weibo.com

乙方： 河北师范大学

地址： 河北省石家庄市南二环东路20号

联系人： 傅学庆

联系方式： 13582101623

联系邮箱： fuxueqing@mail.hebtu.edu.cn

一、定义

本协议各项条款除非另有明确规定，否则下列词语应具有如下含义：

1.1 **微博，**指基于用户关系的信息分享、传播以及获取平台，用户可以通过 PC、手机等多种移动终端接入，以不超过 2000 字的文字，同时辅以图片、视频等多媒体形式，实现信息的即时分享，域名包括但不限于 weibo.com、weibo.com.cn、t.cn 等。

1.2 **开放平台，**即微博开放平台，指由甲方拥有并提供软件、文档和技术服务的平台（URL 包含但不限于：http://open.weibo.com），开发者可基于这些软件、文档和技术服务向用户提供应用服务产品。

1.3 **开放平台规范体系，**指使用微博开放平台必须遵守的一系列协议、管理规范、管理办法、注意事项等，包括但不限于：开发者协议、应用运营管理规范、微连接分级管理办法、应用审核产品指南、应用安全开发注意事项、平台应用设计规范等。开放平台规范体系为本协议不可分割的一部分。

1.4 **商业数据，**即微博商业数据，通过 API（Application Programming Interface，应用程序编程接口）提供，基于特定关键字/用户/微博等，实时推送或主动获取的微博官方数据。

1.5 **商业数据服务，**指甲方通过开放平台商业数据接口为接入方提供获取微博商业数据的付费服务。

1.6 **商业数据服务使用费，**指商业数据服务接入方使用商业数据服务而需支付的费用，具体包括约定的套餐使用费及套餐外使用费等。

1.7 **服务产品，**指商业数据服务接入方利用甲方许可的开放平台商业数据接口和商业数据服务资源等，在甲方许可范围内向其用户提供的产品或其他服务，以下简称“产品”。

1.8 **关联企业**，指一方控制另一方或为另一方所控制或双方共同受控制于同一实体的任何企业。控制是指直接或间接拥有该企业百分之五十（50%）以上的股权、投票权或管理权。

二、合作方式

2.1 甲方向乙方有偿提供商业数据服务，包括但不限于：商业数据接口接入服务、接口使用数据统计服务及其它为履行本协议而应当提供的技术服务；乙方在按期付款并遵守开放平台规范体系相关规定的前提下，可在甲方许可的范围内使用甲方提供的商业数据。

2.2 本协议仅适用于中国大陆及港、澳、台地区，即乙方通过甲方商业数据服务所获取的商业数据仅限于服务中国大陆及港、澳、台地区注册的法人或其他组织。

三、商业数据服务套餐资费

3.1 商业数据服务套餐

[illegible]	[illegible]	[illegible]	[illegible]	套餐外使用费
A	2000 万条	1 年	60,000 元	2.8 元/千条
B	1 亿条	1 年	250,000 元	1.7 元/千条
C	5 亿条	1 年	800,000 元	1.3 元/千条

注：“返回条数”指甲方商业数据接口根据乙方设置的调用参数所返回的数据条数。

3.2 资费说明

3.2.1 套餐使用费：在套餐使用期限内，乙方内容类数据返回条数未超出所购买套餐的条数，按所购买套餐的使用费付费；套餐使用期限内未使用完的内容类数据返回条数视为乙方放弃使用，不可延期使用或要求甲方折价返还。

3.2.2 套餐外使用费：在套餐使用期限内，乙方内容类数据返回条数超出所购买套餐的条数，需按照本档套餐的套餐外使用费对超出部分支付超额使用费。

3.3 费用支付

3.3.1 套餐使用费：乙方应于本协议签署之日起 7 个工作日内将商业数据服务套餐_A_中约定的套餐使用费__60000__元人民币支付到甲方指定的银行账户中，套餐

使用费一旦支付概不退还，甲方在收到乙方上述使用费之后按照本协议约定时间为乙方提供服务，如乙方延期付款的，则甲方有权顺延本协议项下的服务开通时间，甲方无需因此承担任何违约责任。

3.3.2 套餐外使用费：乙方如需使用套餐外数据， 需向甲方提出书面（电子邮件或信函等）申请。超出套餐限量部分的费用，乙方应按照甲方出具的数据使用统计结果向甲方按月支付：即自使用套餐外数据次月起，每自然月 10 日前支付上个自然月的套餐外使用费。

3.3.3 若乙方未按时支付上述套餐使用费或套餐外使用费的，则从该自然月 11 日起（包含 11 日）每日按应付使用费 3‰的比例向甲方支付滞纳金。年度累计 3 次未按期缴费或欠费达 30 个自然日者，除需缴齐费用及滞纳金外，甲方有权终止本协议。

3.4 乙方使用本协议项下服务数据量以甲方提供的数据为准，若乙方对使用数据量有异议且无法协商的，乙方可委托双方认可的具备合法资质的第三方机构进行系统记录审核，甲方应予以配合。在此情况下，以第三方机构审核确认的数据量为准；但除非第三方数据显示甲方存在过错（数据差大于 15%），否则委托该第三方产生的任何费用由乙方独自承担。

3.5 甲方在收到乙方支付的商业数据服务使用费后，根据乙方的书面申请(含电子邮件形式)，在 10 个工作日内向乙方提供合法有效的发票。

3.6 甲方账户信息：

账户：北京微梦创科网络技术有限公司

银行账号：110907172810405

支行名称：招行建国路支行

四、双方权利与义务

4.1 甲方权利与义务

4.1.1 为符合数据使用安全的原则，甲方有权要求乙方在本协议签订前或本协议履行期间提交相关文件或资料，以证明乙方具有履行本协议及合法安全使用商业数据的资格，乙方需在甲方要求的时间内予以提供，否则甲方有权拒绝为乙方提供或继续提供本协议项下商业数据服务。

4.1.2 甲方有权审核乙方所购买商业数据服务的内容和范围，并根据确定的内容和范围向乙方开放相应的数据接口，指导乙方使用，并为数据服务的持续使用进行技术维护和支持。

4.1.3 甲方负责解决本协议项下商业数据服务相关技术问题，如遇系统升级，甲方将免费提供升级服务；若因调整、升级或更新等需要暂停提供商业数据服务，甲方将提前告知乙方；若乙方在使用过程中对商业数据服务有反馈或建议，乙方有权向甲方反映。

4.1.4 为保证乙方对商业数据的使用符合国家相关法律法规的要求，甲方有权对乙方检索、获取、使用商业数据的过程进行监控及跟踪。若乙方在使用商业数据的过程

中存在下列问题，甲方有权在乙方提供合法有效的证明或合理解决之前，暂停继续提供商业数据服务；若乙方未能在甲方要求的期限内合理解决，甲方有权以单方通知的形式终止本协议。若因此给甲方造成任何损失，甲方有权追偿，并保留必要时向相关部门报告或举报的权利：

（1） 违反国家法律法规或相关政策的规定等；

（2） 未获得权利人合法完整授权或侵犯第三方合法权益等；

（3） 违反本服务协议、开放平台规范体系、或微博相关协议或规则等；

（4） 超出授权范围及授权期限使用商业数据、或未及时有效处理纠纷诉讼致使第三方向甲方主张权利等；

（5） 按照国家相关司法、行政主管机构的要求等（此种情况下，甲方有权在不通知的情况下先按照上述要求进行处理）；

（6） 其他导致乙方不具备履行本协议的资质或能力的情况。

甲方上述监控及跟踪并不意味着甲方对乙方在使用商业数据过程中行为的任何承诺或担保，也并不构成甲方与乙方使用商业数据行为的任何连带责任，乙方需对自身的行为独立负责。

4.1.5 对于如下情况，甲方不承担任何责任：

（1） 非因甲方原因造成的服务障碍或损失，如乙方自身或第三方原因，包括但不限于电力、电信、自身软硬件环境、或自身使用不合理等；

（2） 乙方与其他第三方之间产生的任何纠纷或诉讼等。

4.2 乙方的权利与义务

4.2.1 乙方应就商业数据服务的接入和使用自行准备相应的硬件和软件环境，承担相应费用，并确保相关软硬件环境的安全性。甲方对由于乙方硬件或软件环境的故障、不匹配、损毁以及由此引起的商业数据服务使用障碍提供不承担任何责任。

4.2.2 乙方需按照甲方的要求在约定的时间、范围内使用其通过本协议获得的商业数据服务，不得超出甲方授权范围使用本协议项下服务、不得使用本协议项下服务进行任何违反国家相关法律法规或侵犯第三方合法权益（包括但不限于财产权、知识产权等）的活动，并且不得以赠与、售卖、授权等任何方式允许第三人使用。若乙方对使用商业数据服务提供的产品有修改，应以书面形式提前 10 个工作日告知甲方并获取甲方书面同意。

4.2.3 因商业数据的特殊性，乙方通过本协议所获取的数据及服务仅限商业用途和科研项目，且乙方应当提前将该具体用途告知甲方并经甲方同意。

项目说明如下：

项目名称： 基于 VGI 的旅游地理研究

数据用途： 主要用于：1.选取带有地理位置信息的数据用于科学研究：主要利用位置微博数据来分析和挖掘旅游地的时空演变和游客时空行为特征。成果形式为公开发表的学术论文和博士、硕士学位论文。2.为特定旅游地提供互联网旅游数据分析。成果形式为分析报告和地图集。

参与项目的团队主要有:

李仁杰 教授 （身份证号: 130102197507130672 ）

傅学庆 副教授 （身份证号: 130102197710140657 ）

李照航 （身份证号: 130533198912180017 ）

4.2.4 未经甲方书面允许，乙方不得将本协议项下商业数据及服务用于以下用途，包括但不限于：（1）不得用于公关等方面的信息披露；（2）不得用于任何形式的政务舆情监测、分析或与之相关的服务等（包括但不限于支持政府舆情监控、政务舆情类产品开发等）；（3）不得用于影响甲方声誉、甲方产品或服务信誉的任何活动。甲方有权根据自身需要调整前述用途限制范围，乙方承诺，若甲方对前述用途限制范围进行调整，自甲方书面通知之日起，乙方即予以遵守。

4.2.5 乙方需保证对于产品拥有合法、完整的权利（包括但不限于所有权、知识产权或其他相关权利），不违反国家法律法规并不侵犯任何第三方合法权益。若因乙方产品导致任何投诉、诉讼、纠纷等，乙方需自行解决；如因此给甲方造成任何损失，乙方应向甲方承担赔偿责任；如给甲方声誉造成损害，乙方应恢复甲方声誉。

4.2.6 乙方承诺，其产品应提供对甲方和用户隐私及安全的保障，其保障内容应不少于或不低于微博开放平台相关规范中对最终用户各项权利的保障水平。不得侵害用户隐私和数据安全、不得干扰甲方及其关联企业相关平台、产品或服务（包括但不限于微博、新浪网等）的任何部分或功能的正常运行。除双方另有书面约定的情况外，乙方产品应该严格按以下相关内容的要求进行：

【用户隐私及数据安全】

（1）本协议项下的产品需要通过“OAuth 授权”的方式对用户帐号进行操作，不允许存储用户在微博的帐号（电子邮件）和密码；

（2）不允许对外泄漏、售卖或授权用户微博的经营数据、内容、策略以及用户在本协议项下的产品中的行为、数据、帐号等信息；

（3）不允许授权、明示或默示任何第三方将用户在本协议项下的产品中的行为、数据、帐号等信息对外泄露；

（4）如乙方在使用本协议项下数据或服务过程中依据法律规定需要获取相关用户授权同意的，由乙方自行负责向此类用户获取相关授权。

【功能要求】

（1）乙方产品中对微博各类指标定义与微博官方保持一致，并在官方调整后在开放平台规定的时间内完成自身指标的同步更新；

（2）不允许将微博的各类指标、效果等与其他渠道，如搜索等进行对比，避免造成客户理解误差；

（3）乙方产品对微博内功能的定义应与微博原生工具保持命名方式一致，如赞、关注等；

（4）乙方产品程序中不允许有其他违反法律、侵犯甲方或任何第三方合法权益的行为。

4.2.7 乙方不得将本协议约定的权利、义务以任何形式分派、次级许可、或转让给任何第三方（包括乙方关联公司）。由乙方法律身份变化造成相关权利义务必须分派和转让的，必须事先获得甲方书面同意，并保证不对本协议的履行产生任何负面影响。

4.2.8 如乙方违反上述约定，或未按时缴纳费用、未按照甲方要求在合理范围内使用商业数据服务、或从事任何其他有损于甲方及其关联企业的行为，乙方在收到甲方缴纳费用、停止不合理或侵权行为通知之日起 7 个工作日内拒不缴纳或改正的，甲方有权立即对乙方采取以下处理措施，包括但不限于暂停或终止提供本协议项下服务，以单方通知的方式终止协议等，乙方已缴纳的费用将不予退还。对于给甲方造成其他后果的，甲方有权追究乙方法律责任并要求乙方承担甲方的全部损失。

4.2.9 如甲方应用户要求或者根据国家法律法规政策规定，需要对微博数据进行屏蔽或删除等处理的，乙方应在收到甲方通知（通知形式包括但不限于书面、邮件、数据接口推送等）后 12 小时内作出删除处理。乙方违反该约定的，甲方有权立即终止本协议，同时，乙方应按照本协议总金额的 30%向甲方支付违约金，如违约金不足以弥补甲方损失的，乙方还应予以补足。

五、保密条款

5.1 甲、乙双方知悉并了解，除双方在本协议中约定的内容以外，任一方（披露方）都有可能提供并允许其他方（接收方）接触到一些其他技术、财务或其它信息，且该信息被认为对披露方具有商业价值且不被公众知悉（包括本协议内容，统称“保密信息”）。任一方在作为接收方时，应将其自披露方处获得的保密信息进行严格的保管，仅为履行本协议义务的目的而使用保密信息，不得向任何第三方披露、泄露任何保密信息；并应确保其有可能接触到保密信息的雇员、代理同等遵守本协议保密条款的相关规定。

5.2 在本协议终止或披露方任何时间提出要求，接收方应将载有披露方保密信息的任何文件、资料或软件，按披露方要求归还披露方，或予以销毁，或进行其他处置，并且不得继续以任何方式使用这些保密信息。

5.3 若任一方违反前述保密条款的约定而导致披露方受到损害，守约方将有权基于适用法律向违约方主张赔偿。

5.4 本协议终止后，双方在本协议项下的保密义务并不随之终止，双方仍需遵守本协议的保密条款，履行其承诺的保密义务；直至披露方同意解除此项义务，或事实上不会因违反本协议保密条款而给披露方造成任何形式的损害时为止。

六、知识产权

6.1 甲方是本协议项下商业数据及商业数据服务的所有权及知识产权权利人。本协议的签订并不意味着甲方与乙方之间发生任何所有权或知识产权的移转，乙方仅可以在甲方允许的范围内使用本协议项下商业数据及相关数据服务等。

6.2 未经甲方事先书面许可，乙方不得擅自使用甲方的名称、商标、标识、象征物或其他任

何类似文字或图形等。

6.3 为更好地提供服务，乙方同意授权甲方（包括但不限于甲方及其关联企业）在营销宣传（包括但不限网站、电子邮件、产品说明、市场营销等）活动中，使用乙方品牌以及相关产品等各种形象资料。

七、陈述与保证

7.1 双方互相向其他方声明、陈述和保证如下：

其是合法设立并有效存续的公司；

其有资格从事本合同项下之合作；

其有权订立本协议，授权代表也已获得充分授权可代表签署本协议；

其有能力履行其于本协议项下之义务，并且该等履行义务的行为不违反任何对其有约束力的法律文件中的限制；

其并非清算、解散或破产程序的主体。

八、利益冲突

8.1 乙方不得利用商业数据服务牟取不正当利益，不得为牟取不正当利益而对任何甲方或甲方关联企业员工有任何贿赂行为，否则甲方有权随时单方提前终止本合同而无需承担任何责任，乙方并应赔偿甲方因此遭受的全部损失。

8.2 在本协议有效期内，如有任何甲方或甲方关联企业在职员工为乙方的股东或者高级管理人员，乙方必须立即书面通知甲方或甲方关联企业法律部门，否则甲方有权随时提前终止本协议而无需承担任何责任。

九、不可抗力

9.1 不可抗力是指本协议双方不能预见、不能合理控制或即使预见也无法避免的事件，该事件妨碍、影响或延误任何一方根据本协议履行其全部或部分义务。鉴于网络的特殊属性，除自然灾难、战争、政府行为以外，还包括：黑客攻击、计算机病毒侵入或发作；计算机系统遭到破坏、瘫痪或无法正常使用而导致信息或记录的丢失；电信部门技术调整导致之重大影响；因政府管制而造成的暂时性关闭；宽带或其他网络设备或技术提供商的服务延迟、故障或其他类似事件等。

9.2 由于上述不可抗力事件致使本合同部分或全部不履行或延迟履行的，双方彼此不承担任何违约责任。但遭遇不可抗力事件的一方，应于不可抗力事件发生后 15 个工作日内书面通知其他方，并提供相关证明。双方可按照事件对协议履行的影响程度，再行决定是否继续履行本协议或终止协议。

9.3 若不可抗力事件持续 20 日或在本协议有效期内累计超过 30 日的，任一方有权以书面通知的形式单方提前终止本协议。

十、违约责任

10.1 本协议双方应正当正确行使权利，及时履行义务，保证本协议的顺利履行；

何类似文字或图形等。

6.3 为更好地提供服务，乙方同意授权甲方（包括但不限于甲方及其关联企业）在营销宣传（包括但不限网站、电子邮件、产品说明、市场营销等）活动中，使用乙方品牌以及相关产品等各种形象资料。

七、陈述与保证

7.1 双方互相向其他方声明、陈述和保证如下：

其是合法设立并有效存续的公司；

其有资格从事本合同项下之合作；

其有权订立本协议，授权代表也已获得充分授权可代表签署本协议；

其有能力履行其于本协议项下之义务，并且该等履行义务的行为不违反任何对其有约束力的法律文件中的限制；

其并非清算、解散或破产程序的主体。

八、利益冲突

8.1 乙方不得利用商业数据服务牟取不正当利益，不得为牟取不正当利益而对任何甲方或甲方关联企业员工有任何贿赂行为，否则甲方有权随时单方提前终止本合同而无需承担任何责任，乙方并应赔偿甲方因此遭受的全部损失。

8.2 在本协议有效期内，如有任何甲方或甲方关联企业在职员工为乙方的股东或者高级管理人员，乙方必须立即书面通知甲方或甲方关联企业法律部门，否则甲方有权随时提前终止本协议而无需承担任何责任。

九、不可抗力

9.1 不可抗力是指本协议双方不能预见、不能合理控制或即使预见也无法避免的事件，该事件妨碍、影响或延误任何一方根据本协议履行其全部或部分义务。鉴于网络的特殊属性，除自然灾难、战争、政府行为以外，还包括：黑客攻击、计算机病毒侵入或发作；计算机系统遭到破坏、瘫痪或无法正常使用而导致信息或记录的丢失；电信部门技术调整导致之重大影响；因政府管制而造成的暂时性关闭；宽带或其他网络设备或技术提供商的服务延迟、故障或其他类似事件等。

9.2 由于上述不可抗力事件致使本合同部分或全部不履行或延迟履行的，双方彼此不承担任何违约责任。但遭遇不可抗力事件的一方，应于不可抗力事件发生后 15 个工作日内书面通知其他方，并提供相关证明。双方可按照事件对协议履行的影响程度，再行决定是否继续履行本协议或终止协议。

9.3 若不可抗力事件持续 20 日或在本协议有效期内累计超过 30 日的，任一方有权以书面通知的形式单方提前终止本协议。

十、违约责任

10.1 本协议双方应正当正确行使权利，及时履行义务，保证本协议的顺利履行；

10.2 任何一方没有充分、及时履行义务的，应当承担违约责任，给守约方造成经济损失的，应赔偿守约方因此遭受的全部经济损失；

10.3 除本协议另有约定，若任一方违约且在守约方书面通知后七个工作日内仍未改正，守约方有权以单方通知的形式立即终止本协议，终止自守约方通知发出之日起生效；同时，守约方有权要求违约方承担相应的违约及赔偿责任。

十一、争议解决与适用法律

11.1 如任何一方就本协议内容或其履行发生任何争议，双方应进行友好协商；协商不成时，任何一方均可向北京市海淀区人民法院提起诉讼。

11.2 本协议的订立、履行和解释及争议的解决均适用中华人民共和国法律法规。

十二、其它

12.1 本协议经双方盖章后于本协议首页所载日期之日起生效，即有效期限为：2018年10月25日至2019年10月24日。

12.2 除本协议中特别约定的情形外，若一方欲提前终止本协议，须提前 30 日书面通知其他方，终止自通知发出之日起 30 日后生效。本协议提前终止不影响终止前双方根据本协议约定所享有的权利和应当承担的义务。

12.3 若双方欲变更本协议，需另行签署书面补充协议方可生效；本协议的所有附件和补充协议（如有）为本协议的组成部分，与本协议具有同等法律效力。若本协议任何部分被视为无效或不可执行，不影响本协议其他条款或部分的有效性和可执行性。

12.4 本协议一式四份，甲、乙双方各执两份，具有同等法律效力。

12.5 本协议未尽事宜由双方另行协商解决。

（以下无正文，为盖章处）

甲方：北京微梦创科网络技术有限公司　　**乙方：河北师范大学**
